# Effect of smartphone app on post-traumatic stress disorder in COVID-19 convalescent patients: A protocol for systematic review and meta-analysis

**DOI:** 10.1097/MD.0000000000025479

**Published:** 2021-04-09

**Authors:** Yunyan Wang, Xia Yang, Haiyan Chen, Yanfang Xu

**Affiliations:** a Nursing Department; b Department of Gastroenterology; c Department of Orthopedics; d Department of Pneumology, The Sixth People's Hospital of KunShan, KunShan 215321, Jiangsu Province, China.

**Keywords:** Coronavirus Disease 2019, post-traumatic stress disorder, smartphone app, systematic review

## Abstract

**Background:**

The outbreak of Coronavirus Disease 2019 (COVID-19) seriously affects humans’ health worldwide physically and mentally. Studies revealed that the prevalence of post-traumatic stress disorder (PTSD) increased under this condition. PTSD can change the structure of patients’ central nervous system, and increase the risk of anxiety or depression, thus greatly affecting the quality of patients’ life and their families. PTSD is preventable, and the effects of early prevention are better. Non-drug intervention can prevent or reduce the psychological sequelae after hospitalization, help patients understand the experience during hospitalization, and be beneficial to their psychological rehabilitation. Whether smartphone app based intervention can be an alternative therapy for PTSD in terms of COVID-19 convalescent patients is still controversial. Therefore, we conducted a meta-analysis and systematic review to evaluate the effects of smartphone app based intervention on PTSD in COVID-19 convalescent patients, so as to provide some guidance for clinical application.

**Methods:**

The literatures that are related to the smartphone app based intervention and PTSD in COVID-19 convalescent patients from inception to February 2021 will be searched. The following databases are our focused areas: ClinicalTrials.gov, Cochrane Central Register of Controlled Trials repositories, PubMed, EmBase, and Web of Science databases. According to the inclusion and exclusion criteria, 2 investigators would independently screen the literature extract data and evaluate the risk of bias in the included studies. Meta-analysis was performed with RevMan5.3 software.

**Results:**

The results of this meta-analysis will be submitted to a peer-reviewed journal for publication.

**Conclusion:**

The conclusion of our study could provide evidence for the judgment of whether smartphone app based intervention is an effective intervention on PTSD in COVID-19 convalescent patients.

**PROSPERO registration number:**

CRD42021240340.

## 1 Introduction

Coronavirus Disease 2019 (COVID-19) is a global pandemic caused by the Severe Acute Respiratory Syndrome Coronavirus-2 (SARS-CoV-2).^[1–3]^ It has become a key issue and has seriously threatened the public health around the world.^[4]^ The outbreak of this infectious disease has affected people's physical health and mental health, including the infected and not infected. Post-traumatic stress disorder (PTSD) and PTSD-related symptoms are prevalent and disabling conditions occur as a consequence of traumatic events.^[5]^ PTSD refers to a psychological disorder and it occurs late and persists after experiencing horrible or dangerous events.^[6]^

A meta-analysis revealed that the incidence of PTSD during pandemic COVID-19 outbreak is 18%,^[7]^ while the prevalence of PTSD-related symptoms in coronavirus survivors is 29%.^[7]^ The lifetime prevalence rate of PTSD is about 4% that is often irreversible once it occurs.^[8]^ People with PTSD may be accompanied by persistent negative emotions, including anger, guilt, fear, or shame.^[9]^ PTSD can result in structural changes in patients’ central nervous system, and increase the risk of anxiety or depression, thus seriously affecting the quality of patients’ life.^[10]^ If it is not intervened, it will seriously disrupt patients’ daily life.^[11]^

A number of studies have pointed out that PTSD is preventable, and the effects of early prevention are satisfactory.^[12–14]^ The latest PTSD treatment guidelines recommend that patients should receive psychotherapy in the outpatient first.^[15,16]^ However, many factors limit patients’ access to treatment, including the limited number of qualified therapists and the high cost of psychotherapy.^[16]^ In addition, patients are often afraid of discrimination, so they are reluctant to take the initiative to visit a doctor. The use of smartphones is more popular in both developed and developing countries.^[17]^ Smartphone app can provide patients with flexible and convenient treatment, and may also make these patients who have no intention of treatment be willing to receive timely treatment.^[18]^

By 2017, more than 300,000 health-related apps were available to consumers, of which 490 were for mental health and behavioral disorders.^[19]^ Therefore, mobile app may have potential advantages in the improvement of PTSD.

At present, there is no consistent conclusion on whether smartphone app based intervention can reduce the incidence of PTSD in the convalescent stage of COVID-19. Considering the low-cost and easy-to-accept intervention method, smartphone app based intervention has application value in the field of psychological reconstruction during the rehabilitation of COVID-19. Therefore, this study conducted a meta-analysis of the literature on the intervention effects of smartphone app based intervention on PTSD in convalescent patients with COVID-19 to provide objective basis for the intervention of PTSD.

## 2 Methods

### 2.1 Study registration

Our protocol has been registered on the PROSPERO. The registration number is CRD42021240340. We strictly abide by the Preferred Reporting Items for Systematic Review and Meta-Analysis Protocols (PRISMA-P) guidelines.

### 2.2 Inclusion and exclusion criteria for study selection

#### 2.2.1 Inclusion criteria

The inclusion criteria were randomized controlled trials (RCTs), with smartphone app based intervention as the main form of intervention.

#### 2.2.2 Exclusion criteria

1. Repeated publication;
2. Incomplete literature;
3. Nonrandomized controlled trials.

### 2.3 Types of participants

COVID-19 patients who have been clearly diagnosed and now in recovery period, regardless of sex, age, race, or educational and economic status, will be enrolled in the review. Participants must be between 18 and 80 years old.

### 2.4 Interventions and controls

The intervention in the experimental group was based on mobile phone app, and the control was multiple control measures, including blank, placebo, usual or standard care, health education, psychosocial therapy, and drug therapy.

### 2.5 Types of outcome measures

PTSD score after intervention.

### 2.6 Search strategy

The following electronic databases will be searched from inception to February 2021: ClinicalTrials.gov, Cochrane Central Register of Controlled Trials repositories, PubMed, EmBase, and Web of Science databases. As for other sources, we also plan to manually search for the unpublished conference articles and the bibliography of established publications. The search terms on PubMed are as follows: “smartphone∗,” or “app,” and “computer∗” or “phone∗”; COVID-19 (e.g., “Corona Virus Disease 2019” or “Corona Virus”); RCT (“randomized” or “randomly” or “clinical trial”). The combination of Medical Subject Headings (MeSH) and text words will be applied. These search terms are summarized in Table 1.

**Table 1:** Search strategy for PubMed.

Number	Search terms
#1	Web[Title/Abstract]
#2	Online[Title/Abstract]
#3	Internet[Title/Abstract]
#4	App[Title/Abstract]
#5	Application[Title/Abstract]
#6	Computer∗ [Title/Abstract]
#7	E-mail[Title/Abstract]
#8	Phone∗[Title/Abstract]
#9	Smartphone∗[Title/Abstract]
#10	OR/1–9
#11	Stress Disorders, Post-Traumatic[MeSH]
#12	Neuroses, Post-Traumatic[Title/Abstract]
#13	PTSD[Title/Abstract]
#14	Post-Traumatic Stress Disorders[Title/Abstract]
#15	Acute Post-Traumatic Stress Disorder[Title/Abstract]
#16	Chronic Post-Traumatic Stress Disorder[Title/Abstract]
#17	Delayed Onset Post-Traumatic Stress Disorder[Title/Abstract]
#18	Neuroses, Posttraumatic[Title/Abstract]
#19	Posttraumatic Stress Disorders[Title/Abstract]
#20	Stress Disorder, Post Traumatic[Title/Abstract]
#21	Stress Disorders, Posttraumatic[Title/Abstract]
#22	Acute Post Traumatic Stress Disorder[Title/Abstract]
#23	Chronic Post Traumatic Stress Disorder[Title/Abstract]
#24	Delayed Onset Post Traumatic Stress Disorder[Title/Abstract]
#25	Neuroses, Post Traumatic[Title/Abstract]
#26	Post Traumatic Stress Disorders[Title/Abstract]
#27	Post-Traumatic Neuroses[Title/Abstract]
#28	Post-Traumatic Stress Disorder[Title/Abstract]
#29	Posttraumatic Neuroses[Title/Abstract]
#30	Posttraumatic Stress Disorder[Title/Abstract]
#31	Stress Disorder, Post-Traumatic[Title/Abstract]
#32	Stress Disorder, Posttraumatic[Title/Abstract]
#33	Stress Disorders, Post Traumatic[Title/Abstract]
#34	OR/11–33
#35	Corona Virus [Title/Abstract]
#36	Corona Virus Disease 2019 [Title/Abstract]
#37	COVID-19 [Title/Abstract]
#38	Novel coronavirus[Title/Abstract]
#39	Novel coronavirus pneumonia[Title/Abstract]
#40	OR/35–39
#41	Random∗[Title/Abstract]
#42	Clinical trial[Title/Abstract]
#43	OR/41–42
#44	#10 AND #34 AND #40 AND #43

### 2.7 Data collection and analysis

#### 2.7.1 Studies selection and data extraction

**Figure 1:**
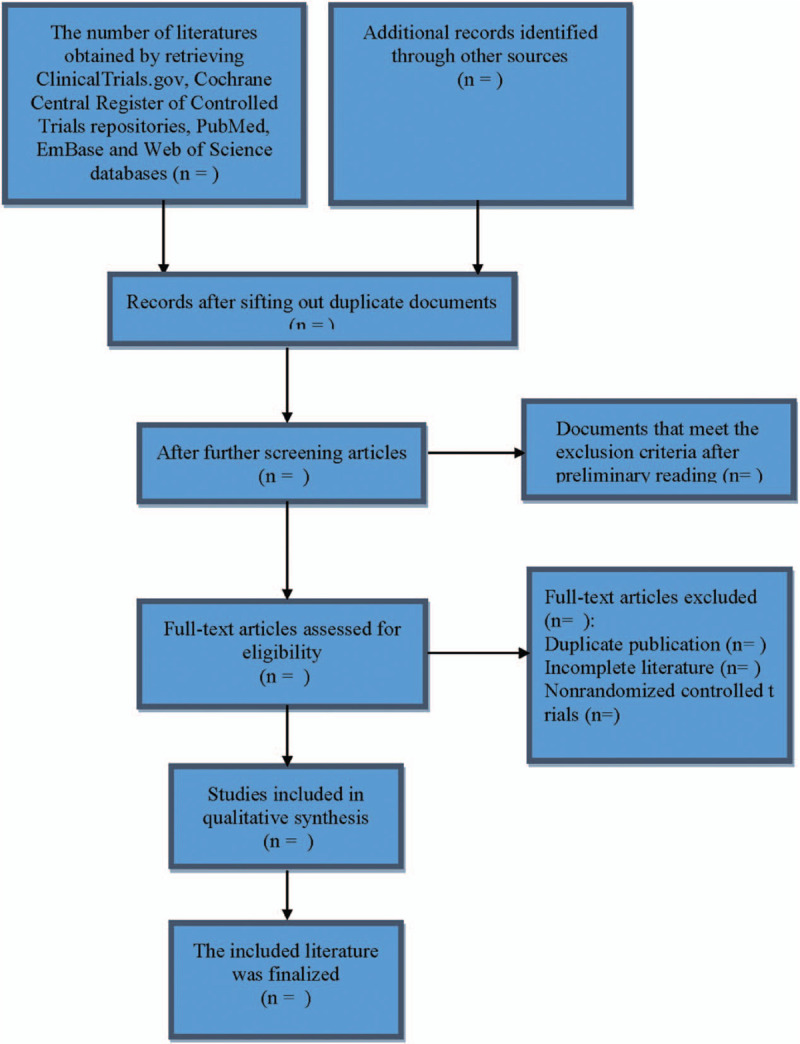
Flow diagram of study selection process.

The details of the selection process will be displayed in the PRISMA flow chart (Fig. 1). First of all, the 2 reviewers will independently check the titles and abstracts of the search results, and initially sift through the articles. After reading the full text of the preliminary selected articles, the 2 independent reviewers will select qualified studies on the basis of our predetermined inclusion criteria. Finally, the articles that are selected by the 2 independent commentators will be integrated after putting forward repetitive parts. When the 2 independent reviewers disagree with each other, a third reviewer will make the decision. Extracted contents include author, publication date, country, sample size, age, type, content and the duration of smartphone app based intervention, PTSD scale, result measurement data, intervention time, and other details.

#### 2.7.2 Assessment of risk of bias in included studies

The risk of bias assessment method set out in the Cochrane Handbook for Systematic Reviews of Interventions will be adopted on the included RCTs.

#### 2.7.3 Measure of treatment effect

Standardized mean difference is applied to measure the efficacy of 95% confidence interval.

#### 2.7.4 Dealing with missing data

The accuracy of the data may affect the results of the study, and even lead to different conclusions. Therefore, we will contact the corresponding authors to supplement the research results with insufficient or missing data. If the corresponding author cannot be contacted, the data will be processed separately, and the potential impacts of incomplete data on the results will be analyzed and reported as well.

#### 2.7.5 Assessment of heterogeneity

The heterogeneity assessment will be conducted by the Review Manager 5.3. According to the Cochrane Handbook, Chi-Squared test and *I*^2^ value could be used to evaluate the heterogeneity. *I*^2^ values of 25%, 50%, and 75% are considered as low, moderate, and high heterogeneity, respectively.

#### 2.7.6 Assessment of reporting biases

If enough studies are available, we will evaluate the funnel plot to report bias.^[20]^

#### 2.7.7 Data synthesis

The Review Manager 5.3 software will be used for the quantitative data analysis, including mapping overall forest plot, heterogeneity analysis, and subgroup analysis. If the *I*^2^ value is less than 50%, it represents that the relative heterogeneity is small, and the fixed effect model should be adopted. Otherwise, the random effect model will be used.

#### 2.7.8 Subgroup analysis

Considering the possibility of a high degree of heterogeneity, we will perform a subgroup analysis, if necessary, to explain the underlying causes of heterogeneity. According to the course of intervention and the content of app intervention, subgroup analysis was carried out.

#### 2.7.9 Sensitivity analysis

Sensitivity analysis will be conducted to assess the robustness of the results. After the data are synthesized, we will exclude the merged studies one by one for sensitivity analysis to see if there exist any significant changes in the comprehensive results.

#### 2.7.10 Quality of evidence

The evidence evaluation of all results will be summarized by the suggested assessment, development and assessment (GRADE) method.^[21]^ The level of evidence will be divided into high, moderate, low, and very low quality.

#### 2.7.11 Ethical review and informed consent of patients

The content of this article does not involve moral approval or ethical review and will be presented in print or at relevant conferences.

## 3 Discussion

The occurrence of PTSD will seriously reduce the life quality of COVID-19 survivors and influence their physical and mental recovery.^[22–24]^ Therefore, it is of great significance to prevent the occurrence of PTSD in survivors of COVID-19. Recently, there has been the emergence of smartphone app based intervention that is increasingly used to treat PTSD. More and more evidence shows that, smartphone app based intervention has positive effects on the life quality, stress relief, and health improvement of patients with PTSD.^[25–29]^ Previous studies have suggested that the intervention of mental illness based on smartphone app is effective.^[30,31]^ Therefore, mobile app may have potential advantages in terms of improving PTSD.

Due to the differences in sample, time, frequency, method, and duration of app intervention, the specific effects of PTSD symptoms may be different. However, it is still necessary to carry out studies with large sample size, and well-designed and long-term follow-up in the future. In addition, future researches should set up a monitoring module in app to objectively record the app login frequency and the duration of the research object, so as to make the research results more accurate, and further explore the impacts of intervention measures based on mobile phone app on PTSD.

## 4 Conclusion

As far as we know, this is the first systematic review and meta-analysis. The findings of this study will prove the effectiveness of smartphone applications in the intervention of PTSD in convalescent COVID-19 patients.

## Author contributions

**Conceptualization:** Yanfang Xu.

**Data collection:** Xia Yang and Yunyan Wang.

**Data curation:** Yunyan Wang.

**Funding acquisition:** Yanfang Xu.

**Funding support:** Yanfang Xu.

**Investigation:** Yunyan Wang.

**Methodology:** Yunyan Wang.

**Project administration:** Yanfang Xu.

**Resources:** Yanfang Xu, Yunyan Wang.

**Software operating:** Yunyan Wang.

**Software:** Yunyan Wang, Xia Yang.

**Supervision:** Xia Yang, Haiyan Chen.

**Validation:** Xia Yang, Haiyan Chen.

**Visualization:** Haiyan Chen.

**Writing – original draft:** Yunyan Wang and Yanfang Xu.

**Writing – review & editing:** Yunyan Wang and Yanfang Xu.
